# Treating colorectal peritoneal metastases with an injectable cytostatic loaded supramolecular hydrogel in a rodent animal model

**DOI:** 10.1007/s10585-023-10210-0

**Published:** 2023-05-22

**Authors:** Anne G. W. E. Wintjens, Hong Liu, Peter-Paul K.H. Fransen, Kaatje Lenaerts, Geert C. van Almen, Marion J. Gijbels, M’hamed Hadfoune, Bas T.C. Boonen, Natasja G. Lieuwes, Rianne Biemans, Ludwig J. Dubois, Patricia Y.W. Dankers, Ignace H.J.T. de Hingh, Nicole D. Bouvy

**Affiliations:** 1 Department of Surgery, Maastricht University Medical Centre, PO Box 5800, Maastricht, 6202 AZ The Netherlands; 2 NUTRIM - School of Nutrition and Translational Research in Metabolism, Maastricht University, Maastricht, The Netherlands; 3 UPyTher BV, Eindhoven, The Netherlands; 4 Department of Pathology, Maastricht University Medical Centre, Maastricht, The Netherlands; 5 Department of Medical Biochemistry, Experimental Vascular Biology, Amsterdam Infection and Immunity, Amsterdam Cardiovascular Sciences, Amsterdam University Medical Center, Amsterdam, The Netherlands; 6 Department of Precision Medicine, Maastricht University, Maastricht, The Netherlands; 7 Institute for Complex Molecular Systems, Eindhoven University of Technology, Eindhoven, The Netherlands; 8 Department of Biomedical Engineering, Laboratory of Chemical Biology, Eindhoven University of Technology, Eindhoven, The Netherlands; 9 GROW - School for Oncology and Reproduction, Maastricht University, Maastricht, The Netherlands; 10 Department of Surgery, Catharina Hospital, Eindhoven, The Netherlands

**Keywords:** Peritoneal metastases model, Colorectal cancer, Intraperitoneal delivery, Injectable supramolecular hydrogel, Mitomycin C, UPy-PEG

## Abstract

Patients with peritoneal metastases (PM) of colorectal cancer have a very poor outcome. Intraperitoneal delivery of chemotherapy is the preferred route for PM treatment. The main limitation of the treatment options is the short residence time of the cytostatic, with subsequent short exposure of the cancer cells. To address this, a supramolecular hydrogel has been developed that allows both local and slow release of its encapsulated drug, mitomycin C (MMC) or cholesterol-conjugated MMC (cMMC), respectively. This experimental study investigates if drug delivery using this hydrogel improves the therapeutic efficacy against PM. PM was induced in WAG/Rij rats (n = 72) by intraperitoneally injecting syngeneic colon carcinoma cells (CC531) expressing luciferase. After seven days, animals received a single intraperitoneal injection with saline (n = 8), unloaded hydrogel (n = 12), free MMC (n = 13), free cMMC (n = 13), MMC-loaded hydrogel (n = 13), or cMMC-loaded hydrogel (n = 13). Primary outcome was overall survival with a maximum follow-up of 120 days. Intraperitoneal tumor development was non-invasive monitored via bioluminescence imaging. Sixty-one rats successfully underwent all study procedures and were included to assess therapeutic efficacy. After 120 days, the overall survival in the MMC-loaded hydrogel and free MMC group was 78% and 38%, respectively. A trend toward significance was found when comparing the survival curves of the MMC-loaded hydrogel and free MMC (p = 0.087). No survival benefit was found for the cMMC-loaded hydrogel compared to free cMMC. Treating PM with our MMC-loaded hydrogel, exhibiting prolonged MMC exposure, seems effective in improving survival compared to treatment with free MMC.

## Introduction

Colorectal cancer (CRC) is the third most common cancer and the second leading cause of cancer-related death worldwide [1, 2]. Death of CRC is most often the result of metastatic disease [3]. After metastasis to the liver, dissemination to the peritoneal cavity occurs most frequently in CRC, causing peritoneal metastases (PM) in patients [4]. PM are most often the result of the intraperitoneal spreading of malignant cells originating from the primary tumor via transmural growth, but it can also occur due to the shedding of cells during surgical removal of the primary tumor [5].

Patients with PM have the worst prognosis compared to patients with distant metastases of CRC [6, 7]. Most PM patients are treated with palliative systemic chemotherapy [8], but the therapeutic response is poor and the survival benefit often remains limited to only several months [6]. It is known that PM have a limited response to systemic chemotherapy compared to other metastatic sites of CRC due to the poor blood supply of the peritoneal surface and hence low penetration of the cytostatic drug into tumor nodules [9, 10]. The local intraperitoneal delivery of a cytostatic agent has pharmacological advantages and has been proposed as an alternative drug delivery route to improve the outcome for patients with PM because the intraperitoneal tumor tissue can be directly exposed to drug concentrations much higher than those that can be achieved with systemic therapy while minimizing systemic toxicity [11]. Today, the standard of care consists of cytoreductive surgery (CRS) followed by hyperthermic intraperitoneal chemotherapy (HIPEC) but is only available for selected patients with limited peritoneal dissemination and no distant metastases [12]. The need for adjuvant HIPEC after CRS was questioned after the recent PRODIGE-7 trial as radical CRS alone has been reported to result in a survival benefit of over 40 months [13–15]. Currently, pressurized intraperitoneal aerosol chemotherapy (PIPAC) is explored as an experimental method to offer local palliative treatment for PM patients with inoperable disease. However, the exposure time of tumor nodules to the cytostatic agent remains limited in both HIPEC and PIPAC which reduces the therapeutic efficacy [16]. Consequently, methods that prolong local drug exposure are needed to improve the outcomes of intraperitoneal chemotherapy.

In previous years, research has been devoted to the development of drug delivery systems (DDSs), aiming to extend the cytostatics’ intraperitoneal residence time without causing systemic toxicity [17]. Previously, we have demonstrated the intraperitoneal use of a hydrogel based on poly(ethylene glycol) polymers functionalized with ureido-pyrimidinone moieties (UPy-PEG). The hydrogel is pH-sensitive; it can be intraperitoneally injected as a liquid while transforming into a hydrogel in situ. The hydrogel has been shown to form a homogeneous soft layer over the visceral- and parietal peritoneum [18]. Furthermore, UPy-functionalization creates hydrophobic domains in the hydrogel which can facilitate the encapsulation of hydrophobic drug substances [19, 20].

In the present study, the UPy-PEG hydrogel is loaded with cytostatic agents and applied for the intraperitoneal delivery of chemotherapy. Mitomycin C (MMC) is the most frequently used agent for HIPEC in patients with colorectal PM [21, 22]. Recently, we demonstrated that a comparable supramolecular hydrogel MMC formulation (h-MMC) showed release of MMC in vitro in 24 h [19]. To provide a more sustained, controlled release, we modified the MMC molecule with cholesterol (cMMC), thereby increasing MMC’s affinity for the hydrogel’s hydrophobic compartments and serving as an anchor to keep the drug retained within the hydrogel [19, 20]. This resulted in the cMMC hydrogel. In vitro release data of cMMC showed an initial burst release of 7% on the first day, followed by a steady controlled release of 1% per day over the following two weeks [19].

In the current experiment, the therapeutic efficacy of hydrogel-formulated MMC (h-MMC) and cMMC (h-cMMC) is evaluated in vivo in a PM rat model to assess the added benefit of hydrogel-induced sustained drug delivery over intraperitoneal delivery of the ‘naked’ unformulated MMC and cMMC molecules.

## Materials and methods

### Formulation of hydrogel, free MMC, and free cMMC

The UPy-PEG polymer was synthesized under aseptic conditions in a comparable way as previously described [23–25]. Polymer powder (SupraPolix, Eindhoven, the Netherlands) was sterilized using ethylene oxide gas. To obtain 1 mL of a 6 wt% UPy-PEG hydrogel, 60 mg polymer powder was added to 0.94 mL autoclaved PBS (pH 11.7) and was stirred at 70 °C until dissolved. After cooling down to room temperature, the pH was adjusted to 8.5-9.0 with sterile filtered (0.2 μm) 1 M NaOH or HCl.

MMC (Beta Pharma, Shanghai, China) was added from 52 mM stock solution in a sterile filtered (0.2 μm) PBS 7.4. Cholesterol was coupled to MMC as previously described [19]. cMMC was added from a 52 mM stock solution in sterile-filtered DMSO. MMC solution for administration was prepared by diluting the 52 mM stock solution into sterile-filtered PBS 7.4 until the final concentration of 0.2 mM MMC. cMMC solution for administration was prepared by dilution of the 52 mM stock solution into sterile-filtered PBS 7.4 with 5% ethanol until the final concentration of 0.2 mM. h-cMMC was left stirring overnight at 4 °C to solubilize the cMMC, whereas the other formulations dissolved readily while stirring. Formulations were loaded into 5 mL syringes and stored at 4 °C overnight prior to administration. The hydrogel formulations and free drug-containing solutions were administered at a volume-to-weight ratio of 20 mL/kg, corresponding to 3–5 mL hydrogel or solution per animal. MMC and h-MMC were administered at a dose of 10 mg/m^2^ (= 1.36 mg/kg), based on the literature [26]. For example, in an animal weighing 250 g, 5 mL of the hydrogel or free drug solution was administered, containing 0.34 mg MMC. The cMMC and h-cMMC dose was the molecular equivalent of 10 mg/m^2^ (= 7.6 mg/kg). Again, 20 mL/kg h-cMMC or c-MMC solution was administered, containing 1.9 mg cMMC in an animal of 250 g.

### Ethics

The study protocol (AVD1070020198765) was approved by the ethical committee of animal experiments, which complied with the Dutch Animal Experimental Act, and was approved by the Animal Experimental Committee of Maastricht University. Data reporting of this experiment followed the ARRIVE (Animal Research: Reporting of In Vivo Experiments) guidelines.

### Animals and housing

Male and female WAG/Rij rats (body weight 140–280 g, 12 weeks old) were purchased from a registered breeding company (Charles River Laboratories, Calco, Italy). The animals were socially housed in filtertop cages (two to three animals per cage) in a temperature- and humidity-controlled room with 12-hour light/dark cycles. Access to non-acidified drinking water and food (Ssniff, Bio Services Uden, The Netherlands) was *ad libitum*. The acclimatization period was at least one week. All animal procedures were conducted at the animal center of Maastricht University. The WAG/Rij strain was chosen because this is a commonly used strain in animal models for PM [27].

### In vitro cell culture and transduction process

PM was induced with the syngeneic colon adenocarcinoma CC531 cells (purchased at CLS Cell Lines Service GmbH, Eppelheim, Germany, product number 500,387). The cells were cultured in RMPI 1640 medium (Thermo Fisher Scientific) supplemented with 10% fetal bovine serum (Sigma-Aldrich, Zwijndrecht, the Netherlands) in a 5% CO_2_/air incubator at 37 °C.

CC531 cells were transduced with a neomycin-resistant firefly luciferase gene, as previously described [28]. Briefly, lentiviral particles were produced by transfecting HEK 293FT with the vectors pLenti PGK V5 luciferase (Addgene plasmid #21,471), pVSV-G, and pCMVd8.74. CC531 cells were transducted with these lentiviral particles, resulting in CC531-Luc cells. Selection with neomycin 2 mg/ml (Sigma-Aldrich geneticin G418, Zwijndrecht, the Netherlands) began 48 h after the transduction process to isolate the resistant colonies positively tested for luciferase activity. After at least three weeks of continuous selection, cells were used in experiments. Before injecting into the animals, the cells were tested for the absence of mycoplasma and rat-specific viral pathogens.

### In vivo PM model

Four animals were intraperitoneally inoculated with 2 × 10^6^ CC531-Luc cells suspended in 1 mL RPMI as described previously [29]. The body weight and welfare were monitored daily. Based on the literature, a macroscopic tumor load on the seventh day after inoculation was expected [30, 31], which was confirmed in two animals by bioluminescence imaging (BLI) and macroscopic evaluation after euthanasia. In addition, two animals were monitored until the endpoint on day 23 to study the dissemination and consequences of untreated PM, including the development of ascites, clinical symptoms related to intraperitoneal tumor growth, and BLI signal development.

### Therapeutic efficacy study

A total of 72 animals were inoculated as described. Seven days later, the animals were randomized to one of the six intervention groups receiving a single intraperitoneal injection with saline (n = 8), empty/unloaded hydrogel (n = 12), free MMC (n = 13), free cMMC (n = 13), MMC-loaded hydrogel (h-MMC) (n = 13), or cMMC-loaded hydrogel (h-cMMC) (n = 13).

Administration of the intervention was performed as previously described [18]. In short, under isoflurane anesthesia, the treatment was administered intraperitoneally in the lower right quadrant. The first seven days after administration of the intervention, paracetamol (200 mg/kg, Dafalgan, Bristol-Myers Squibb, Belgium) was provided via drinking water.

### Bioluminescence imaging and analysis

Tumor progression and treatment efficacy were monitored weekly until euthanasia, via BLI. Animals were anesthetized with isoflurane and D-luciferin (150 mg/kg, Perkin Elkmer, Rotterdam, the Netherlands) was administered intraperitoneally. White light and BLI images were acquired from a ventral position ten minutes after luciferin administration using an iXon Ultra 897 camera (Andor Technology Ltd., Belfast, United Kingdom) in the X-Rad 225Cx machine (Precision X-ray, Inc, North Branford, CT, USA) using no filters (open modus). Signal intensity was calculated using ImageJ; the cumulative raw BLI signal intensity was obtained after subtracting the background signal measured at a standardized area outside of the abdomen.

### Study outcomes

The primary endpoint of this study was overall survival. Secondary endpoints were tumor progression quantified by weekly BLI, intraperitoneal tumor load at sacrifice, ascites volume at sacrifice, presence of intraperitoneal macroscopic abnormalities at sacrifice, changes in body weight, and clinical symptoms induced by the disease model evaluated by welfare monitoring.

Body weight and welfare were recorded at least three times a week. Welfare was scored by an experienced bio-technician blinded to the assigned group. Whether the humane endpoint (HEP) was reached was based on standardized clinical signs related to intraperitoneal tumor progression. These signs included the presence of massive hemorrhagic ascites (indicated by an increased body weight of 10–20% within several consecutive days, development of pale ears due to anemia, or development of a purple scrotum in male animals), signs of intraperitoneal tumor growth with invalidating consequences, and/or discomfort not responding to analgesia or other supportive treatments. On day 120 after tumor inoculation, the remaining animals were euthanized and autopsied.

For euthanasia, in anesthetized animals, a midline laparotomy was made. All abdominal ascites was collected using a syringe. The animal was euthanized via a terminal cardiac puncture. The abdominal cavity was inspected for abnormalities caused by the hydrogel. The intraperitoneal tumor load was scored semi-quantitatively by calculating the adjusted Peritoneal Cancer Index (PCI) as described before [30, 32]. In short, the abdomen was inspected at eleven regions (injection site subcutaneous, injection site intraperitoneal, omentum, liver hilum, spleen, mesentery, gonadal left and right fat pads, diaphragm, and parietal peritoneum). At each region, tumor load was scored based on the largest tumor size in each region; ranging from 0 (no macroscopic tumor) 1 (limited, diameter 1–2 mm); 2, (moderate, diameter 2–4 mm); to 3 (abundant; diameter > 4 mm or > 10 nodules). The sum of all scores represented the PCI.

### Statistical analyses

Statistical analysis and visual representation of data were computed by GraphPad Prism version 8. Overall survival was defined as animals not reaching a HEP based on intraperitoneal tumor progression. There was a drop-out in animals receiving any hydrogel formulation (n = 4 h-MMC; n = 1 unloaded hydrogel; n = 1 h-cMMC; two animals showed signs of stomach bleeding, in the other animals no abnormalities were found during autopsy) and because of eccentric tumor locations (subcutaneous and intra- thoracically, n = 3 in free cMMC group), or too deep anesthesia (n = 2 in saline group). These animals were excluded from all analyses. The percentage of body weight change was calculated by subtracting the daily measured weight from the baseline weight of each animal. Group comparison of mean body weight was performed with mixed-effect models with post-hoc Dunnett’s correction. Survival analysis was performed using the Kaplan-Meier method and analyzed by the log-rank test. Median PCI were compared between the experimental groups with a two-way analysis of covariance (ANOVA) test with post-hoc Tukey’s multiple comparison test. Correlation was tested by calculating Pearson’s r. The level of significance was set at p < 0.05.

## Results

### Tumor inoculation and the effect of the intervention on body weight

All 72 animals were inoculated with CC531-Luc cells. Clinical conditions and mean body weight did not significantly differ between the groups before intervention administration.

**Fig. 1:**
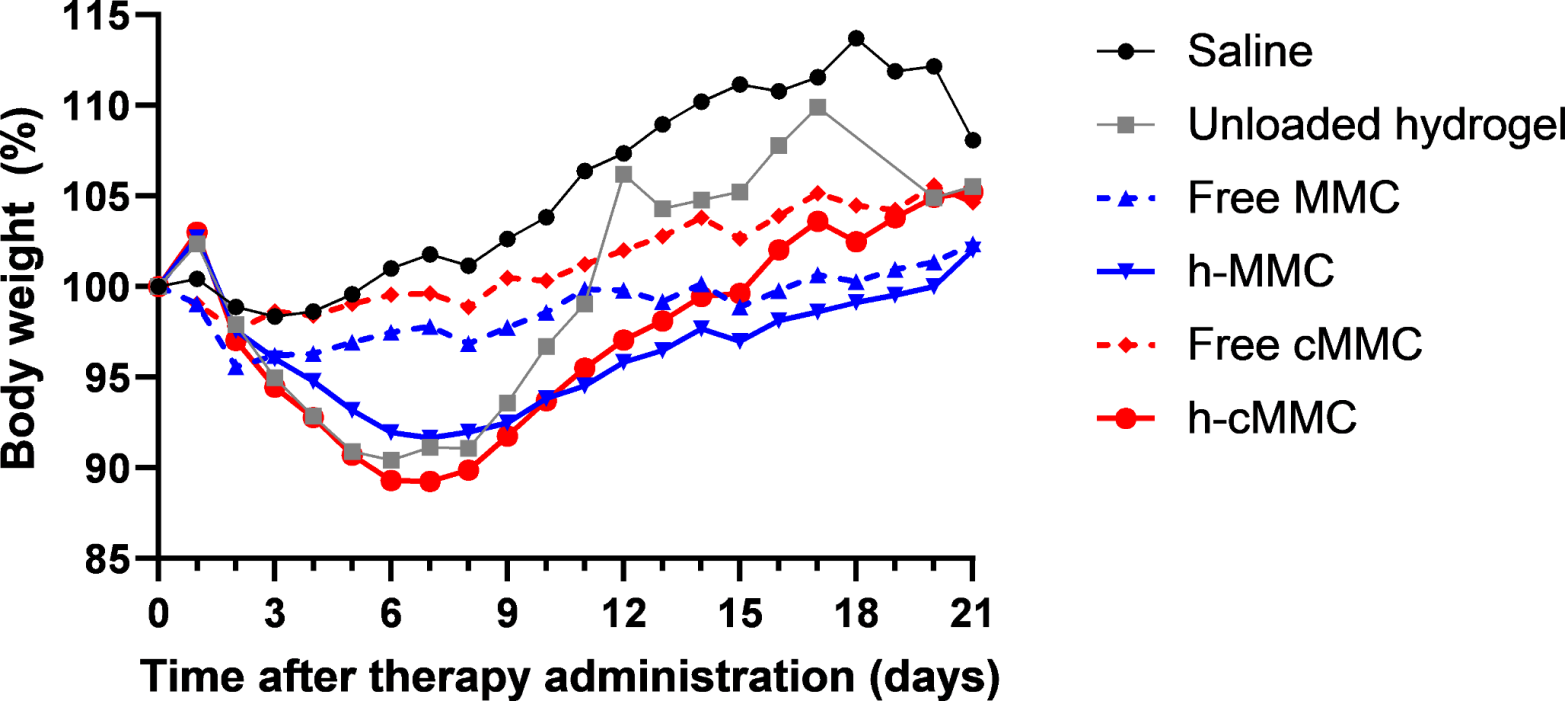
The course of mean relative body weight during the first three weeks after intervention administration

Figure 1 shows the mean body weight for all intervention groups in the first three weeks after administration. In animals in the saline group, stable mean body weight was observed until around day 10 after saline administration. Thereafter, the mean body weight increased, most probably caused by ascites formation and intraperitoneal tumor development, which is a characteristic consequence of intraperitoneal CC531-inoculation [29]. Short before reaching the HEP, a decrease in body weight was often observed indicating clinical progression of the disease (cachexia).

Animals that had hydrogel administered (unloaded hydrogel, h-MMC, or h-cMMC), had an initial weight gain on day 1 related to hydrogel administration, followed by weight loss during the rest of the first week. From day 7, recovery to mean baseline weight was observed in all hydrogel groups, although the time to recover was longer in the h-MMC and h-cMMC groups than in the unloaded hydrogel group. The weight gain in the unloaded hydrogel group continued, with the highest mean body weight on day 17, followed by weight loss.

This trend in body weight was less pronounced in animals in the free MMC- or free cMMC groups. In both groups, the course of the body weight was comparable.

### Overall survival

**Fig. 2:**
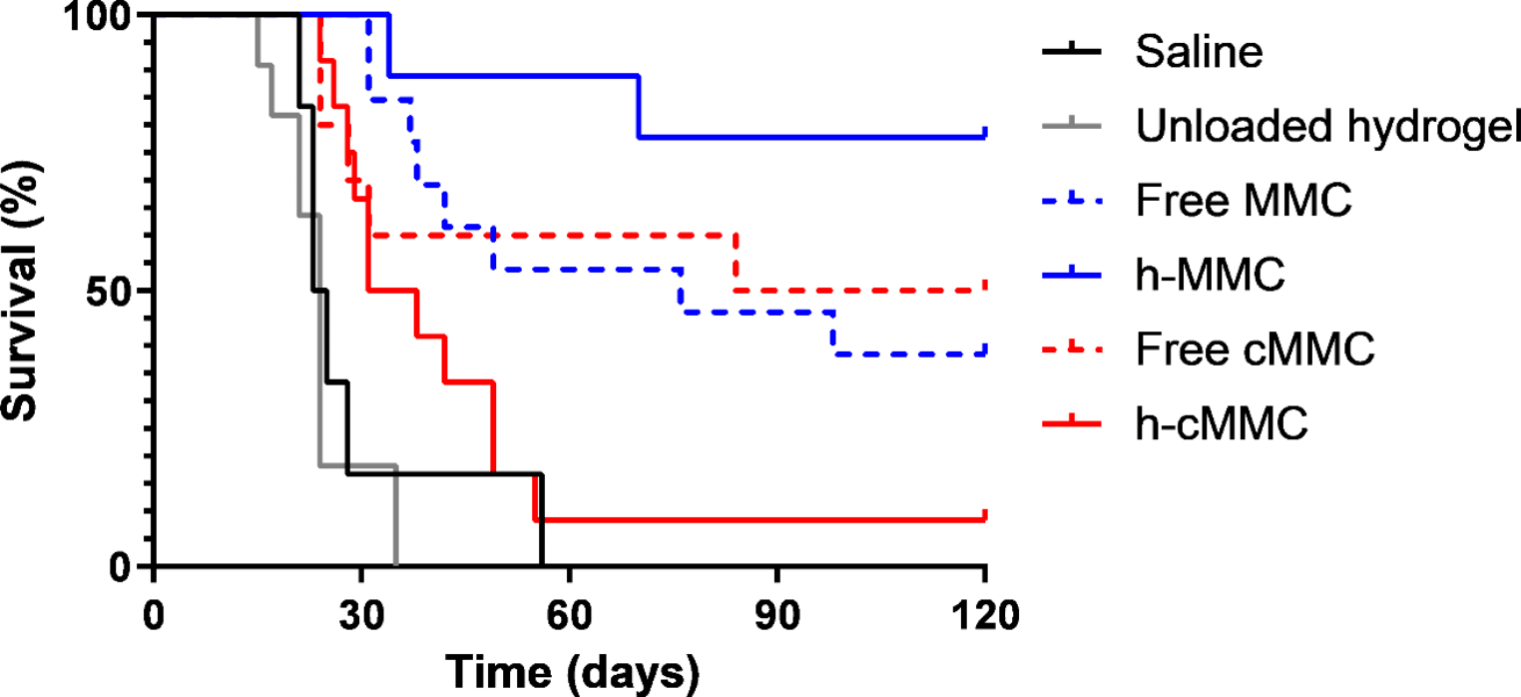
Survival curves of animals in different intervention groups

After completing the 120-day follow-up period, 78% of animals were still alive in the h-MMC group, whereas this was 50% in the free cMMC, 38% in the free MMC, and 8% in the h-cMMC groups. In only one animal that completed the follow-up period (h-MMC group), residual tumor tissue was found during the autopsy. To evaluate the treatment efficacy of h-MMC and h-cMMC, a Kaplan-Meier survival analysis was performed (Fig. 2).

As shown there, the median survival of rats treated with h-MMC was not reached, which was significantly longer when compared to animals not treated with a cytostatic in the saline group (24 days, p < 0.001) or in the unloaded hydrogel group (24 days, p < 0.001). A trend for h-MMC survival benefit was observed when compared to free MMC (p = 0.087).

The median survival of rats treated with h-cMMC was 34.5 days, which was significantly longer compared to the unloaded hydrogel (p < 0.001), but not significant compared to the saline group and free cMMC group (102 days).

Again, h-MMC showed improved survival compared to free MMC, while for cMMC the opposite is observed compared to h-cMMC.

### Macroscopic evaluation and PCI scores after euthanasia

**Fig. 3:**
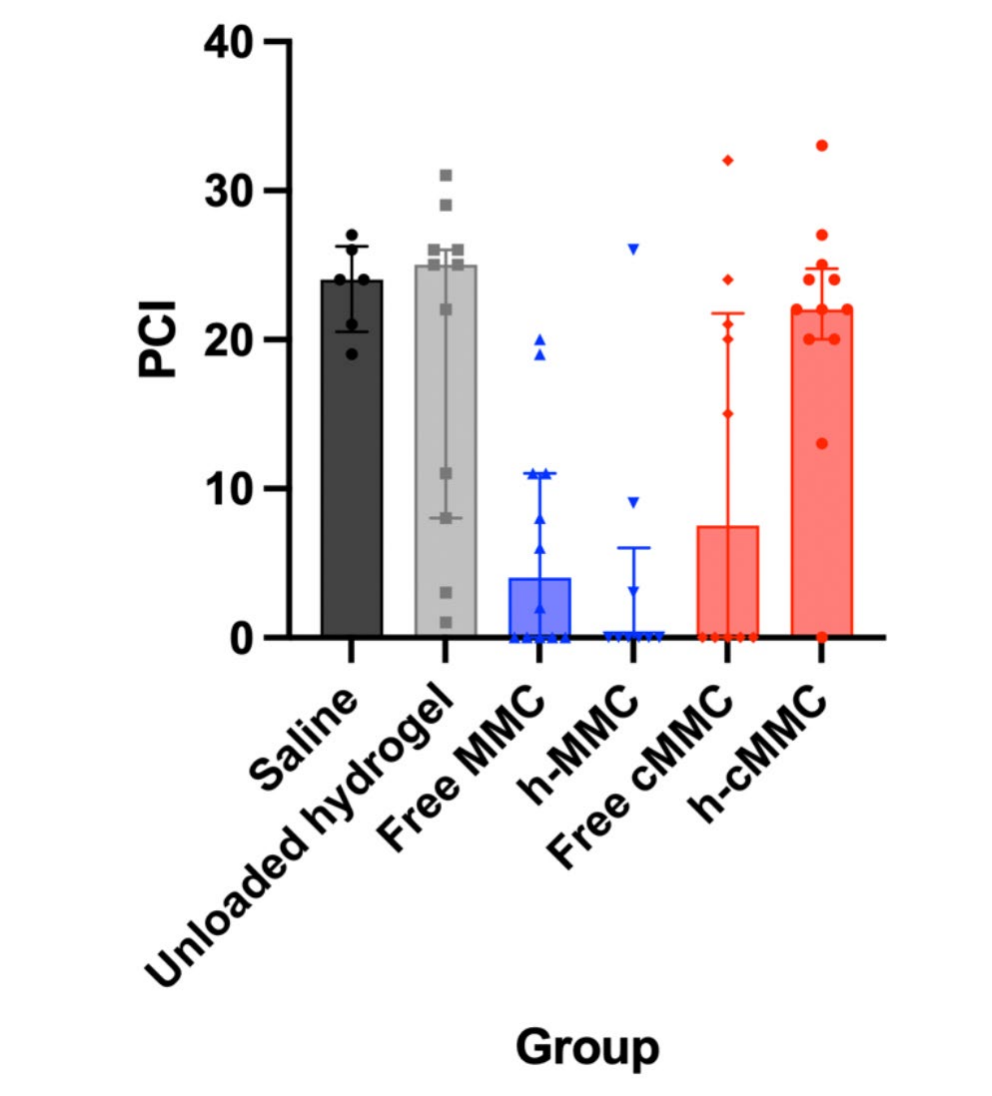
Median PCI scores with IQR per intervention group

Animals’ euthanasia was directly followed by autopsy and macroscopic evaluation. At sacrifice, we did not find remnants of hydrogel or evidence of intraperitoneal abnormalities such as bowel obstruction, caused by hydrogel administration in any of the animals. Subsequently, the PCI was scored; median PCI scores and interquartile ranges (IQR) were calculated and displayed in Fig. 3. The median PCI in the h-MMC group was 0 ± 6, which was significantly lower compared to animals not treated with a cytostatic in the saline group (p = 0.0017) and unloaded hydrogel group (p = 0.0162). The median PCI in the free MMC group was 6 ± 11, which was also significantly lower compared to the median in the saline group (p = 0.0035) and unloaded hydrogel group (p = 0.0271). No significant differences were found in comparing free MMC with h-MMC or free cMMC and h-cMMC.

### Non-invasive monitoring via longitudinal imaging

**Fig. 4:**
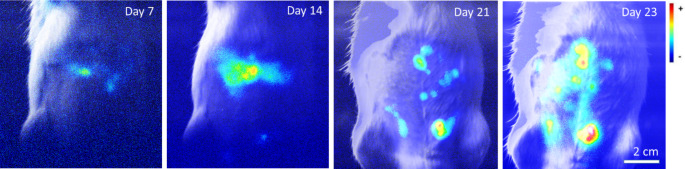
Intraperitoneal development of tumor growth measured by BLI over time in a representative animal in the saline group

All animals in the saline- and unloaded hydrogel group developed BLI-detectable tumor load; the signal intensity increased continuously from day 7 onwards indicating tumor development. In Fig. 4, intraperitoneal tumor growth expressed as signal intensity is shown over time in an animal in the saline group.

**Fig. 5:**
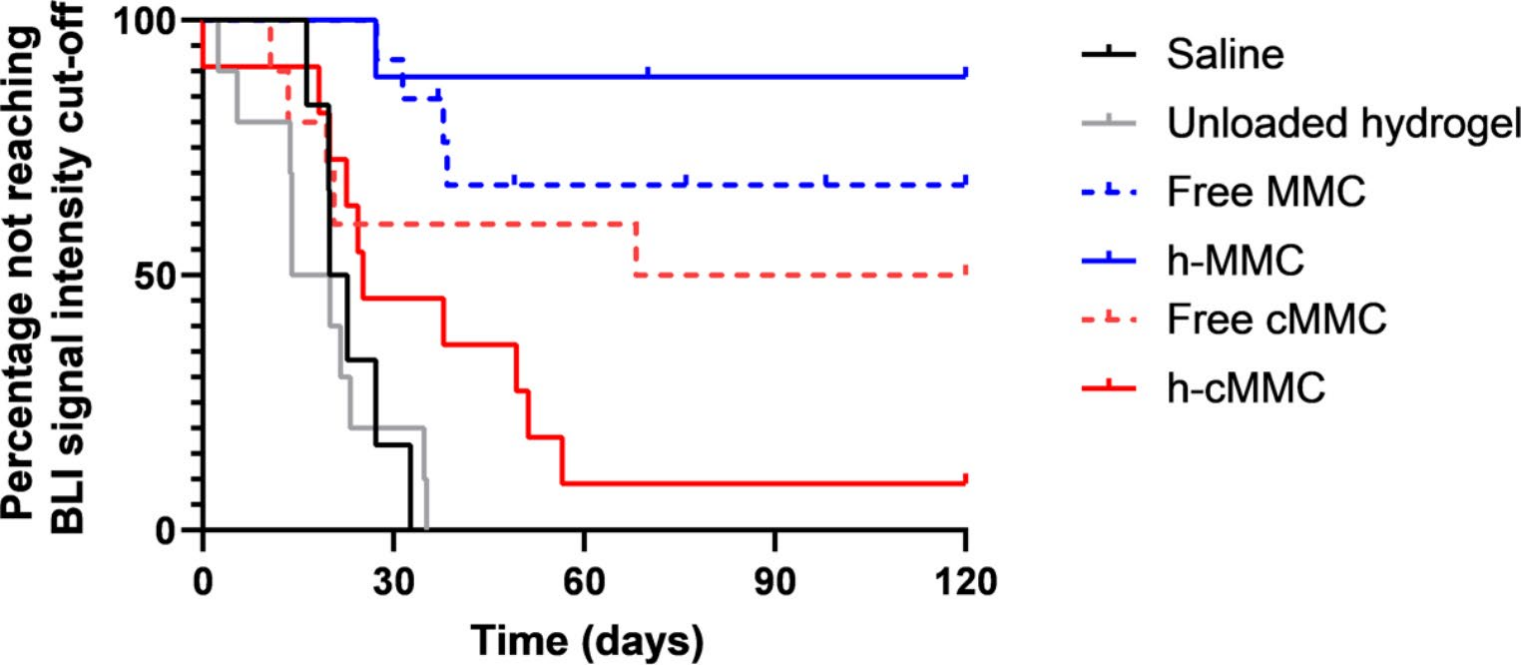
Kaplan-Meier curves are based on not reaching a cut-off signal intensity value measured by non-invasive monitoring via BLI

To investigate if the BLI signal intensity correlates with the progression of tumor development over time, a cut-off value was calculated based on a signal intensity that 80% of the animals not treated with a cytostatic agent - in the saline- and unloaded hydrogel group - had reached during their follow-up. Animals in the saline- and unloaded hydrogel group quickly developed a severe disease burden indicative of imminent death. Figure 5 displays the Kaplan-Meier curves of animals not reaching the cut-off value, which serves as a virtual endpoint. The median time to reach cut-off for the saline-, unloaded hydrogel, free cMMC, and h-cMMC was 21, 17, 94, and 25 days, respectively. In the free MMC and h-MMC groups, the median time to reach the cut-off value was not reached.

The median time to reach this cut-off value in the h-MMC group was not reached, whereas this was significantly shorter in the saline group (21 days, p = 0.0002), unloaded hydrogel group (17 days, p < 0.0001), and h-cMMC group (25 days, p = 0.0005). Between h-MMC and free MMC (median survival undefined) and between h-cMMC and free cMMC (94 days) there were no significant differences.

The therapeutic efficacy measured by BLI is higher for h-MMC compared to free MMC, whilst for free cMMC compared to h-cMMC the contrary is found.

### Correlations between signal intensity & PCI and ascites volume & PCI

**Fig. 6:**
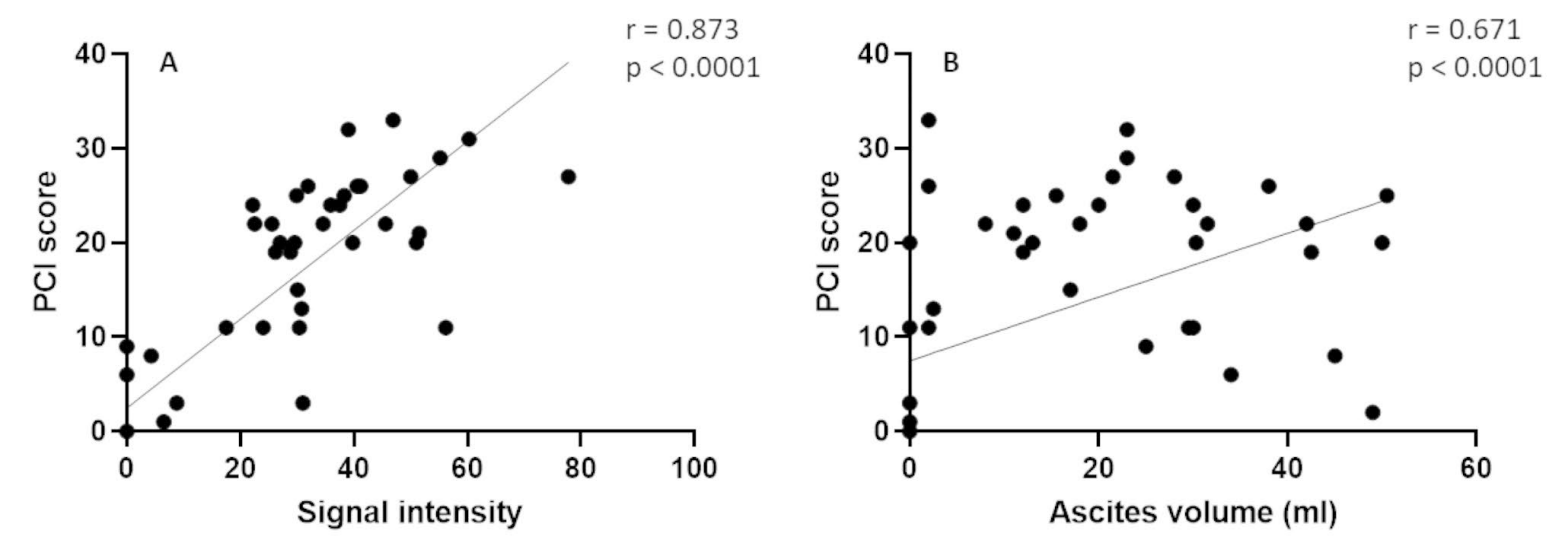
(A) Significant correlation between PCI score and final BLI signal intensity. (B) Significant correlation between PCI score and ascites volume

Prior to sacrifice at HEP, BLI signal intensity was measured followed by macroscopic assessment of the PCI score and ascites volume during necroscopy. Figure 6 indicates a significant correlation between PCI score and final BLI signal intensity (r = 0.873, 95% CI 0.788–0.926, p < 0.0001). In addition, a significant correlation was found between PCI score and ascites volume (r = 0.671, 95% CI 0.483–0.799, p < 0.0001), demonstrating that this PM model is characterized by massive ascites formation, mainly at the terminal phase of the disease.

For animals that reached HEP with a rapid increase in body weight in their disease end phase, the final BLI signal intensity was often lower than the increased signal intensity over time (data not shown). In these animals, a high ascites volume (> 30 mL) was found during autopsy.

## Discussion

We investigated the therapeutic efficacy of prolonged intraperitoneal exposure to mitomycin C (MMC) or cholesterol-conjugated MMC (cMMC) released from a supramolecular hydrogel, administered in a well-known and validated in vivo experimental PM rat model [27, 29] with a distribution pattern similar to human PM [29]. The results were compared to a single injection of the respective free, unformulated drugs. Best survival was obtained in animals treated with MMC formulated in the hydrogel (h-MMC), as 78% of these animals survived the 120-day follow-up period, compared to 38% in the free MMC group. No survival benefit was found for animals treated with h-cMMC compared to free c-MMC.

In current clinical practice, intraperitoneal delivery of a cytostatic is the preferred route for PM treatment. The available treatment options have a major therapeutic drawback: the intraperitoneal residence time is short. During HIPEC, MMC is removed from the peritoneal cavity after 90 min [33], whereas during PIPAC, the therapeutic capnoperitoneum is usually terminated after 30 min [34]. Chemotherapy located intraperitoneally enters the tumor tissue by passive diffusion [35], so prolonged direct exposure to the cytostatic might result in improved tumor penetration and hence increased apoptosis of the cells [11]. A simple extension in HIPEC and PIPAC exposure times is impossible because this will result in systemic peak exposure and consequent systemic toxicity. To increase the intraperitoneal exposure of a high-dose cytostatic with limited systemic uptake, cytostatic-loaded DDS have been developed [17]. Although various studies have described their therapeutic efficacy in experimental PM models [17], there are problems involving toxicity against the carrier or administration of the DDS, hampering clinical implementation so far.

The injectable hydrogel formulation used in the present study, formulated specifically for intraperitoneal administration, has been proven safe and feasible to administer and showed good tissue compatibility after 28 days. Intraperitoneal exposure to the hydrogel did not result in macroscopic adverse effects or signs of organ damage [18]. In the present experiment, no intraperitoneal abnormalities due to the presence of the hydrogel were found during autopsy, confirming the biocompatibility of the hydrogel. The hydrogel’s encapsulated drug is released sustainably, as demonstrated with a comparable hydrogel formulation [19], which encompasses that only a single administration of the hydrogel already results in clinically relevant overall survival benefit. Other systems often require multiple dosing regimens. Due to the homogenous distribution of the hydrogel, the entire peritoneal cavity is exposed to the cytostatic agent for a prolonged period, further enhancing the interaction between the drug and tumor tissues.

In experiments with other DDS, a large variety of cytostatic agents was used, such as 5-FU, paclitaxel, and doxorubicin [17]. These cytostatic agents have their drawbacks. Paclitaxel is a hydrophobic drug that enables a long retention time in the DDS, but after its release in the abdominal cavity, it might be difficult to distribute and reach the tumors [36]. 5-FU, on the other hand, will be released from a DDS more rapidly due to its good solvability but is a cell cycle-specific drug requiring repeated or continuous administration [37–39]. Interestingly, none of these experiments used MMC as a cytostatic agent, although this is - together with oxaliplatin - the most common chemotherapeutic agent used for HIPEC in patients with PM of colorectal origin [22]. MMC is an antitumor antibiotic inhibiting DNA replication by forming crosslinks between the two arms of the DNA double helix [40, 41]. As MMC is more hydrophilic than most cytostatic agents, it will be released more rapidly from the DDS [19]. MMC has several beneficial features. It is not cell-cycle specific, so it does not require repeated administration. It has a good antitumor effectivity against colorectal cell lines. The depth of tissue penetration is several millimeters [42]. Lastly, the systemic uptake of MMC is limited with a short half-life of 60 to 90 min [43]. All together makes MMC the cytostatic agent of choice with significant clinical relevance to study in our experimental PM model.

Due to the hydrophilic nature of MMC, > 80% of the drug was released within the first 24 h from a similar UPy-hydrogel formulation evaluated in vitro [19]. To overcome this initial burst release, we modified MMC with cholesterol (cMMC) creating a novel, more hydrophobic molecule with enhanced retention in the hydrogel. In vitro experiments indicated that cytotoxicity was preserved for h-cMMC [19]. However, it is unknown if the cytotoxicity is preserved in vivo, as the anticipated sustained release of cMMC in the form of h-cMMC did not provide a therapeutic benefit for h-cMMC. The intraperitoneal pharmacokinetics of cMMC are currently unknown. Free cMMC showed comparable therapeutic responses to free MMC, indicating that the interaction of cMMC with hydrogel might lead to impaired therapeutic efficacy of h-cMMC in vivo. Furthermore, the cholesterol modification could increase the affinity of the drug compounds to other macromolecules with a hydrophobic domain, such as albumin. For similar cholesterol-conjugated drugs, it has been described that the drugs bind to albumin and end up in lymph nodes [44, 45]. So, cMMC may bind to abundant albumin molecules in the peritoneal cavity, preventing it from reaching the desired site of action. We may conclude that cholesterol modification has an important impact on the pharmacokinetic behavior of the drug compound.

This experiment has several strengths. It is the first study using an injectable MMC-loaded hydrogel for experimental PM treatment. Choosing MMC improves the clinical relevance over other cytostatic agents. With a follow-up period of 120 days, a good representation of the overall survival benefit of prolonged MMC exposure from the hydrogel is given. Lastly, the therapeutic efficacy of the intervention was non-invasively monitored over time via BLI. A strong correlation was found between PCI score and final BLI signal intensity, indicating that BLI is a suitable tool for the non-invasive measurement of tumor progression. However, there are also some limitations. The main limitation of this experiment is the considerable drop-out in animals shortly after administration of the (un)loaded hydrogel and before therapeutic efficacy could be assessed. This might be caused by the treatment in combination with a rather invasive PM rat model. Second, intraperitoneal inoculation with CC531 cells is associated with the formation of excessive peritoneal fluid, which might lead to quenching of the BLI signal in animals during their terminal phase of disease where large volumes of ascites accumulate. The quenching could be due to the large volume of fluid blocking the signal and/or due to the dilution of the luciferase enzyme (< 1 ml luciferin vs. 30–50 ml ascites). Therefore, future study groups using BLI as a measurement tool to monitor tumor progression over time should focus more on the increasing trend over time of the signal rather than solely the final signal intensity before HEP. Third, we did not measure the parameters of bone marrow suppression, the main adverse side effect related to (repeated) administration of MMC. In humans, the nadir is expected to be at 4–8 weeks post-administration, with recovery at 8–10 weeks, whereas in mice, it was demonstrated that the bone marrow was recovered 7 weeks after repeated MMC administration [46]. As our primary outcome was survival after 120 days, it was expected that possible bone marrow suppression would already be recovered. Fourth and final, the therapeutic efficacy of the hydrogel has only been investigated in a rat PM model using a single syngeneic cell line and has not been confirmed in PM models using other (xenograft) cell lines.

The results of this experiment form a good basis for further research. The first step is investigating the pharmacokinetics of MMC released from the hydrogel, aiming to demonstrate the sustained release principles that are hypothesized to be the key to success in this animal experiment. Because only limited macroscopic tumor deposits were found after the seven-day inoculation period [26, 30], we cannot conclude if a more advanced stage of PM also has therapeutic benefits when treated with h-MMC. Future research is needed in which a longer inoculation period produces a greater disease burden. Future studies might also focus on administrating the h-MMC directly after CRS and compare this with CRS and HIPEC, to investigate if HIPEC can be replaced by perioperative h-MMC administration. In addition, as CRS often involves bowel resection with a consequent anastomosis, future studies should focus on the safety of anastomotic healing with intraperitoneal h-MMC. Finally, PM can develop in the months or years after the removal of the primary colorectal tumor, called metachronous PM. Future studies should explore if administration of h-MMC after resecting of the primary colorectal tumor can help to reduce the incidence of metachronous PM.

## Conclusion

We demonstrated that the treatment with MMC formulated in a supramolecular hydrogel – exhibiting prolonged peritoneal exposure to MMC - contributes to a clinically relevant improved survival compared to treatment with free MMC in WAG/Rij rats with colorectal PM. Animals treated with h-cMMC yielded no survival benefit.

## Data Availability

The datasets generated during and/or analyzed during the current study are available from the corresponding author on reasonable request.
